# Analysis of the associations between moderate-to-vigorous physical activity and screen time on psychological symptoms among university students: a cross-sectional survey based on six geographic regions in China

**DOI:** 10.1186/s12888-024-05945-3

**Published:** 2024-07-16

**Authors:** Guo-feng Deng, Yuan Wen, Jun Cheng, Bo Huang, Ningling Liu

**Affiliations:** 1 School of Physical Education, Nanchang Institute of science & technology, Jiangxi Nanchang, 330000 China; 2 School of Physical Education, Shangrao Normal University, Jiangxi Shangrao, 334000 China; 3 Sports Health and Industrial Development Research Center, Key Research Base of Philosophy and Social Sciences of Jiangxi Province, Shangrao Normal University, Jiangxi Shangrao, 334000 China

**Keywords:** Psychological symptoms, MVPA, Screen time, University students, Associations

## Abstract

**Background:**

Declining physical activity and increasing screen time (ST) among Chinese adolescents have become major concerns shared by scholars, while mental health issues are also on the rise. Previous studies have confirmed the association between physical activity and screen time and psychological symptoms, but it is unclear how their psychological symptoms, especially for Chinese university students who have a high proportion of psychological symptoms, and no research evidence has been found.

**Methods:**

This study investigated physical activity, screen time, and psychological symptoms in 11,173 university students aged 19–22 years in six regions of China. A binary logistic regression analysis was used to analyze the association between moderate-to-vigorous physical activity (MVPA) and screen time and psychological symptoms. And the generalize linear model (GLM) analysis was used to further analyze the association between MVPA and screen time and psychological symptoms.

**Results:**

The detection rate of psychological symptoms among Chinese university students was 16.3%, with a higher percentage of female students (17.5%) than male students (14.7%). The proportion of male students (8.2%) with MVPA > 60 min/d was higher than that of female students (2.3%), and the proportion of male students (33.8%) and female students (34.5%) with screen time > 2 h/d was basically the same. The generalize linear model (GLM) analysis showed that university students with MVPA < 30 min/d and screen time > 2 h/d (*OR* = 1.59, 95% CI: 1.10–2.31) had the highest risk of psychological symptoms (*OR* = 1.59, 95% *CI*: 1.10–2.31) compared to university students with MVPA > 60 min/d and screen time < 1 h/d as the reference group. The risk of psychological symptoms was the highest among those with MVPA < 30 min/d and screen time > 2 h/d (*OR* = 1.59,95% *CI*: 1.10–2.31). In addition, university students with MVPA > 60 min/d and a screen time of 1–2 h/d (*OR* = 0.09, 95% *CI*: 0.03–0.25) had the lowest risk of psychological symptoms (*P* < 0.001). The same trend was observed for both male and female students.

**Conclusion:**

Chinese university students have a certain proportion of psychological symptom problems, and there is a significant between MVPA and screen time and psychological symptoms, and the same trend exists for both male and female students. Chinese university students should perform MVPA for not less than 60 min a day, and at the same time control the duration of screen time, and screen time should be controlled between 1 and 2 h a day, which has a better promotion effect on psychological health.

## Background

In recent years, the issue of university students’ mental health has received extensive attention and research from scholars. The university stage, because of the multiple pressures of family, academics and employment, has led to university-age adolescents being highly susceptible to various mental health problems. Such as depression, anxiety, non-suicidal self-injurious behavior and other adverse mental health problems [1]. The occurrence of these problems has an extremely negative impact on the quality of life, academic performance, and general well-being of university students. It is estimated that one fifth of the world’s university students currently suffer from various types of mental illnesses, but unfortunately 40% of these students do not seek help for the causes and externalities that lead to mental health problems that have a more serious impact on them [2]. In addition, a study of U.S. university students from 2007 to 2018 revealed that depression, anxiety, non-suicidal self-harm, suicidal ideation, and suicide attempts among university students have increased significantly over the past years, showing a doubling in several indicators [3]. The results of a national survey of French university students showed that the prevalence of suicidal thoughts, severe distress, high perceived stress, severe depression and high anxiety among university students was 11.4%, 22.4%, 24.7%, 16.1% and 27.5%, with 42.8% of them reporting at least one mental health problem [4]. A meta-analysis also showed that the prevalence of depression and communication among university students was 33.6% and 39.0%. Among them, the prevalence of depression and anxiety in low- and middle-income countries was 42.5%, 54.2% [5]. The mental health of Chinese university students is also facing serious challenges and the situation is very unpromising. A survey confirmed that 72.84% of university students in China have poor mental health, of which there are gender differences, with females (51.19%) being higher than males (48.81%), and significant differences depending on their place of residence and parents’ education [6]. However, this situation was exacerbated during the past COVID-19 pandemic, which had a more detrimental impact on university student health. Studies confirm that 71.26% of university students in the United States during the COVID-19 pandemic reported that their stress and anxiety levels increased during the pandemic [7]. China also has 45% of university students with more serious mental health problems [8]. In Bangladesh, 28.5% of university student participants also had severe psychological stress, 33.3% had anxiety problems, and 46.92% had depression problems [9]. In conclusion, the mental health problems of university students are facing serious forms and challenges.

Among the factors that affect university students’ mental health, there is a strong association between MVPA time and university students’ mental health. A study confirmed that an increase of 1 h per week in physical activity time among adolescents was associated with an 8% decrease in their rate of depression [10]. Another follow-up study also showed that adequate physical activity time for adolescents is better at reducing the incidence of depression, anxiety and other mental illnesses [11]. An evaluation of adults in the UK, Ireland, New Zealand and Australia showed a strong association between physical activity and mental health, and that increased physical activity facilitates mental health promotion [12]. A randomized controlled trial showed that aerobic exercise had a moderate to large effect on adolescent attention (Standardized mean differences, SMD = 0.84), hyperactivity (SMD = 0.56), and impulsivity (SMD = 0.56) and related symptoms such as anxiety (SMD = 0.66), executive functioning (SMD = 0.58), and social impairment (SMD = 0.59) had moderate to large effects, and physical activity improved the core symptoms of attention deficit hyperactivity disorder [13]. However, physical activity and adolescent mental health levels do not show an absolute linear relationship. A study confirmed that there is a range of physical activity to promote the development of mental health, with 3–5 times a week for 30–60 min each time being optimal for the development of mental health in exercisers, and exercise time > 90 min/d having a negative impact on mental health due to excessive exercise or fatigue [14]. This suggests that future research should also investigate more evidence to delve deeper into the association that exists between physical activity and mental health.

In recent years, with the continuous development of electronic information technology, the frequency and time of using various electronic products in life have gradually increased, which brings a continuous rise in screen time and has a negative impact on mental health. A survey of adolescents in 12 countries showed that the proportion of adolescents’ daily screen time > 2 h/d reached 54.2% [15]. The results of a nationally representative data from 25 countries show that 53.9% of people do not participate in any form of exercise and nearly 40% of people have screen time > 2 h/d [16]. Epidemiological surveys have shown that an increase in adolescent screen time will bring about increased sedentary time, decreased physical activity, increased risk of overweight and obesity, decreased offline social time, and adverse effects on mental health [14, 16]. Studies have confirmed that for every 1 h increase in TV viewing or total screen time, adolescents have a 1.64 and 1.58 times higher risk of depression in adulthood, respectively [17]. However, there are also studies that show the positive effects of reasonable screen time on mental health. Research confirms that the ability of adolescents to use online tools to build friendships and engage in communication activities such as offline real-life gatherings is beneficial for stress release and mental health development [18]. This shows that there is still some disagreement between the relationship between screen time and mental health.

Given the above studies, it can be seen that previous studies have focused on simply analyzing the association that exists between MVPA or screen time and psychological symptoms. Unfortunately, few studies have comprehensively analyzed between MVPA and screen time in relation to psychological symptoms. One study showed that screen time was positively associated with anxiety, depression, and stress in university students, and that prolonged screen time was detrimental to mental health. However, this study also confirmed that outdoor physical time (“green time”) reduced stress and depression levels, but not anxiety levels, and that green time played a moderating role in the association between screen time and mental health [19]. It can be seen that, in addition to the simple association between MVPA and screen time and psychological symptoms, there may be between MVPA and screen time that has a joint effect on psychological symptoms. In view of the above findings, in order to further understand the association between MVPA and screen time and psychological symptoms of Chinese university students. We conducted a survey on 11,173 university students in six regions of China to investigate the association between of MVPA and screen time and the psychological symptoms of university students. We aimed to provide help for mental health promotion and intervention for Chinese university students, and also to provide reference and basis for government departments and universities to develop public health policies and educational systems.

## Methodology

### Participants

The participants of this study were extracted in four stages. Stage 1: Based on the geographical distribution of different provinces in China and taking into account the six geographical divisions of China, Jilin in northeast China, Jiangxi in north China, Anhui in east China, Hubei in south-central China, Sichuan in southwest China, and Xinjiang in northwest China were selected as the survey areas of this study. Stage 2: In each province, taking into account both liberal arts and science universities, two universities were selected as the tested university students. Stage 3: In each university student, taking into account the distribution of liberal arts and science colleges, one randomly selected from each of the arts and sciences colleges and one randomly selected from each of the science colleges. Stage 4: In each college, among the first year to the fourth year of university students, one teaching class was selected randomly and in whole groups for each grade, using the class as the whole group sampling unit.

**Fig. 1:**
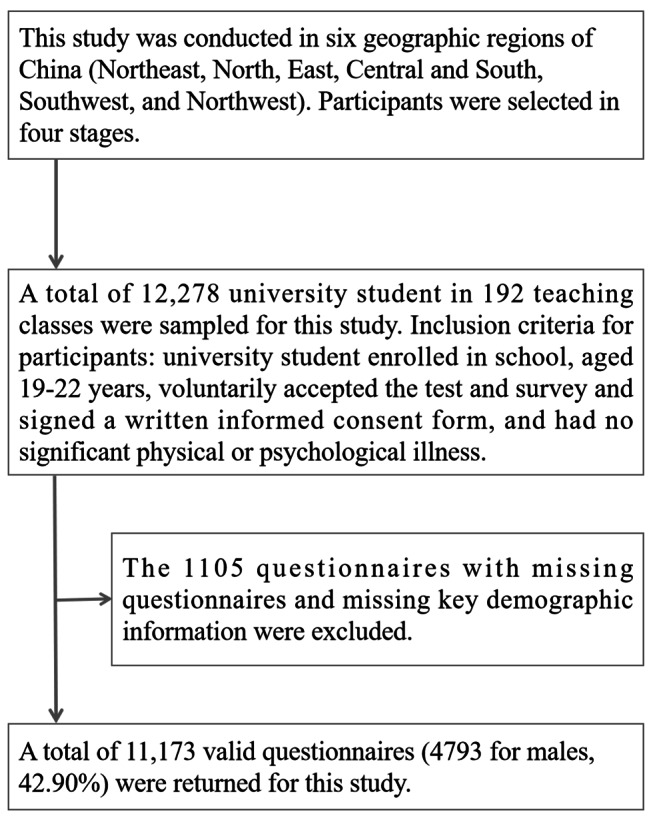
Sampling process of Chinese university student participants

A total of 12,278 university students from 192 teaching classes were selected from six areas of China for this study. After excluding 1105 questionnaires with missing questionnaires, response rates above 80%, and missing key demographic information, a total of 11,173 valid questionnaires were returned (4793 for male students, 42.90%). All 11,173 questionnaires contained valid data on physical activity and screen time. In this study, mean substitution was performed for missing values. The inclusion criteria for the participants in this study were: university students enrolled in school, aged 19–22 years, voluntarily accepted the test and survey and signed a written informed consent form, and no major physical or psychological illness. Informed consent was obtained from the students themselves prior to the investigation of this study, and this research investigation was approved by the Human Ethics Committee of Shangrao Normal University (202,214,045). The specific sampling process of participants in this study is shown in Fig. 1.

### Physical activity

The physical activity survey was conducted using the International Physical Activity Scale (IPAQ) short form, which is commonly used internationally [20]. The questionnaire has been widely adopted among Chinese youth [21, 22]. The IPAQ short form consists of 7 main entries that investigate the number of times participants engaged in physical activities of different intensities in the past 7 days and the average duration of each. For example, walking, lifting heavy objects, and participating in physical exercise. The average number of hours of physical activity per day was calculated based on the number and duration of physical activity over the past 7 days. Based on the recommended standard for Moderate-to-Vigorous Physical Activity (MVPA) of 150 min of moderate physical activity per week, or 75 min of vigorous physical activity per week, or an equivalent [23–26]. This study focused on the analysis of MVPA in university students and divided them into three groups of < 30 min/d, 30–60 min/d, and > 60 min/d for analysis.

### Screen time

In this study, the survey of university students’ screen time was divided into 2 items, namely, “the amount of time they used or watched computer, tablet, cell phone and TV on weekdays in the past 7 days” and “the amount of time they used or watched computer, tablet, cell phone and TV on weekends in the past 7 days “The questionnaire was filled out by university students according to their actual situation. The students filled in the questionnaire according to their actual situation and calculated the average daily screen time in the past 7 days. Reference was made to Chinese scholars [27] and the American Academy of Pediatrics (AAP) classification criteria for screen time (2001). In this study, the screen time was divided into three groups < 1 h/d, 1–2 h/d, and > 2 h/d, respectively.

### Psychological symptoms

The survey of psychological symptoms was conducted using the multidimensional sub-health questionnaire of adolescents (MSQA) developed by the study of Tao Fangbiao et al. [28, 29]. The scale is widely used among Chinese adolescents and has good reliability and validity, Cronbach α coefficient in previous study was 0.963 [29]. The scale consists of 15 items, mainly including “often feel nervous”, “often feel bitter”, and “always feel that most people cannot be trusted”. Each item was selected according to the participants’ actual situation in the past 1 month, and the duration of each problem was chosen as “lasting more than 3 months”, “lasting more than 2 months”, “lasting more than 1 month “, “lasted more than 2 weeks”, “lasted more than 1 week”, “did not last or lasted less than 1 week”, in order that is, the options were 1–6 in order, and the subjects If the participant chose 1–3, the score was recorded as 1, and if the participant chose 4–6, the score was recorded as 0. The questionnaire scores were 0–15. A total score of ≥ 7 was considered as having psychological symptoms, i.e., mental health problems. The questionnaire was divided into three dimensions: emotional symptoms, behavioral symptoms, and social adaptation difficulties, consisting of 7, 4, and 4 items, respectively. A score of ≥ 4 for emotional symptoms, ≥ 1 for behavioral symptoms, and ≥ 2 for social adaptation difficulties indicated a positive result for this dimension.

### Basic information and covariates

The investigation of basic information mainly included the information of participants’ province, region, school, major, class, and age. The survey of covariates included indicators of single-child status, father’s education, mother’s education, socioeconomic status (SES), sleep quality, SSBs, BMI, etc. Only child was classified as “No The only child was categorized as “No” or “Yes”. The father or mother’s education level was classified as “Junior high school and below”, “High School”, “College and above”. “The SES survey uses internationally used indicators such as parental education, parental occupation, and family income to measure the socioeconomic status of adolescents’ families. In this study, SES was calculated by referring to the 2003 Program for International Student Assessment (PISA) [30]. Based on the total SES score of the participants, they were divided into three groups according to percentile: Low (< *P*25), Medium (*P*25-75), and High (> *P*75). Sleep quality was investigated using the Pittsburgh Sleep Quality Index (PSQI) [31]. The scale consists of 7 factors with scores ranging from 0 to 21. In this study, the PSQI scores were used to classify sleep quality into Good (≤ 5), Moderate (6–7), and Poor (> 7) [31]. The sugar-sweetened beverages (SSBs) were used to investigate the participants’ consumption of sugary drinks in the past 7 days, including sugary juices, coffee, functional drinks, carbonated drinks, etc. [32]. In this study, SSBs were classified as ≤ 1 times/week, 2–5 times/week, and ≥ 6 times/week. Body Mass Index (BMI) was calculated based on participants’ height and weight by the formula: BMI = weight (kg)/height (m)^2^. The study was divided into Emaciation, normal, overweight, and obesity four groups based on WHO classification criteria(2006). Height and weight tests are measured according to the testing methods and instruments required by the China Student Physical Fitness Survey [33]. The height was accurate to 0.1 cm and the weight was accurate to 0.1 kg.

### Quality control

The survey consisted of trained teachers. Communication with the school before the survey, students were gathered in classrooms in advance, and the staff entered the classrooms one by one to distribute the online questionnaires by smart devices. Staff explained the purpose and requirements of the survey to students before the questionnaires were filled out. The questionnaires are uniformly distributed and then filled out uniformly. The questionnaires were collected immediately after they were filled out. When the questionnaires were collected, the completion of the questionnaires was checked, and students were asked to add the questionnaires that were missed or wrongly filled in. The questionnaires were filled in anonymously by number. The tests of height and weight were conducted by trained specialists.

### Statistical analysis

Since there were some differences between genders in psychological symptoms, MVPA and screen time, the data were processed and analyzed by gender in this study. The covariates, basic conditions and psychological symptoms of Chinese university students of different genders were expressed as percentages. The psychological symptoms and the status of each dimension among university students with different MVPA and different screen time were expressed as percentages, and the comparisons were made by chi-square test.

The association of MVPA and screen time with psychological symptoms and each dimension was performed by binary logistic regression analysis. Logistic regression analysis by gender was performed with the presence of poor psychological symptoms as the dependent variable and MVPA and screen time as the independent variables. Model 1 was the crude model. Model 2 was adjusted for age, only child or not, father’s education level, mother’s education level, and SES on the basis of Model 1. Model 3 adjusted for sleep quality, SSBs, and BMI on the basis of Model 2.

The association of MVPA and screen time with psychological symptoms was analyzed using generalize linear model (GLM). The odds ratio (OR) and 95% confidence intervals (95% CI) of the results were presented separately.

Statistical analyses were processed by Statistical Package for the Social Sciences 25.0 software (IBM, Armonk, NY, USA) and plotted using Graph Pad Prism 8.0.2 software (Graph Pad Software, Inc., CA). *p* < 0.05 was considered a statistically significant difference by two-tailed test.

## Results

In this study, 11,173 university student aged 19–22 years in six geographic regions of China were surveyed, of which 4793 (42.90%) were male students. Table 1 shows that a higher proportion of male students (30.6%) than female students (22.2%) had “Good” sleep quality and a higher proportion of female students (66.5%) than male students (58.6%) had “Poor” sleep quality.

Among university students with sugar-sweetened beverages ≥ 6 times/week, the percentage of male students (23.5%) was higher than that of female students (16.7%). The proportion of male students (8.2%) with MVPA > 60 min/d was higher than that of female students (2.3%). The proportion of university students with screen time > 2 h/d was basically the same between male students (33.8%) and female students (34.5%). The detection rate of psychological symptoms among Chinese university students was 16.3%, among which the percentage of female students (17.5%) was higher than that of male students (14.7). In terms of each dimension, the detection rates of emotional symptoms, behavioral symptoms and social adaptation difficulties among Chinese university students were 17.5%, 17.9% and 14.6%, respectively.

**Table 1:** Status of covariates, MVPA, screen time and psychological symptoms among university students of different genders in China

Items	Male	Female	Total
**Number**	4793	6380	11,173
**Only child**			
No	3205(66.9)	5173(81.1)	8378(75)
Yes	1588(33.1)	1207(18.9)	2795(25)
**Father’s education**			
Junior high school and below	1457(30.4)	1718(26.9)	3175(28.4)
High School	2984(62.3)	4110(64.4)	7094(63.5)
College and above	352(7.3)	552(8.7)	904(8.1)
**Mother’s education**			
Junior high school and below	2231(46.5)	2889(45.3)	5120(45.8)
High School	2314(48.3)	3219(50.5)	5533(49.5)
College and above	248(5.2)	272(4.3)	520(4.7)
**SES**			
Low	852(17.8)	945(14.8)	1797(16.1)
Medium	3289(68.6)	4581(71.8)	7870(70.4)
High	652(13.6)	854(13.4)	1506(13.5)
**Sleep quality**			
Good	1467(30.6)	1419(22.2)	2886(25.8)
Moderate	515(10.7)	721(11.3)	1236(11.1)
Poor	2811(58.6)	4240(66.5)	7051(63.1)
**SSBs**			
≤ 1 times/week	2685(56)	3637(57)	6322(56.6)
2–5 times/week	980(20.4)	1676(26.3)	2656(23.8)
≥ 6 times/week	1128(23.5)	1067(16.7)	2195(19.6)
**BMI**			
Slimmer	504(10.5)	1370(21.5)	1874(16.8)
Normal	2492(52)	3431(53.8)	5923(53.0)
Overweight	925(19.3)	483(7.6)	1408(12.6)
Obese	872(18.2)	1096(17.2)	1968(17.6)
**MVPA**			
<30 min/d	3216(67.1)	5303(83.1)	8519(76.2)
30–60 min/d	1183(24.7)	931(14.6)	2114(18.9)
>60 min/d	394(8.2)	146(2.3)	540(4.8)
**Screen Time**			
<1 h/d	1461(30.5)	1877(29.4)	3338(29.9)
1–2 h/d	1712(35.7)	2301(36.1)	4013(35.9)
>2 h/d	1620(33.8)	2202(34.5)	3822(34.2)
**Psychological symptoms**			
Emotional symptoms	758(15.8)	1194(18.7)	1952(17.5)
Behavioral symptoms	800(16.7)	1205(18.9)	2005(17.9)
Social adaptation difficulties	706(14.7)	923(14.5)	1629(14.6)
Psychological symptoms	703(14.7)	1119(17.5)	1822(16.3)

Note: SES, socioeconomic status; SSBs, sugar-sweetened beverages; BMI, body mass index; MVPA, moderate-to-vigorous physical activity. SES: Low(< P25), Medium(P25-75), High(> P75);Sleep quality: Good, ≤ 5 points; Moderate, 6–7 points; Poor, >7 points

The results in Table 2 show that the detection rate of psychological symptoms among Chinese university students showed a decreasing trend as the duration of MVPA increased. The detection rate of psychological symptoms among university students with MVPA < 30 min/d was 18.6%, and the detection rate of psychological symptoms among university students with MVPA > 60 min/d was 7.4%, and the difference between groups was statistically significant (χ^2^ = 144.078, *P* < 0.001). In terms of screen time, Chinese university students showed an increasing trend of psychological symptoms with the increase of screen time. The difference was statistically significant (χ^2^ = 233.727, *P* < 0.001) when comparing between groups. The comparison of the detection rate of each dimension of psychological symptoms by gender is shown in Table 2.

**Table 2:** Univariate analysis of different MVPA and screen time with psychological symptoms and factors among Chinese university students

Gender/Category	Group	Number	Emotional symptoms				Behavioral symptoms				Social adaptation difficulties				Psychological symptoms		
			*N* (%)	χ^2^-value	*P*-value		*N* (%)	χ^2^-value	*P*-value		*N* (%)	χ^2^-value	*P*-value		*N* (%)	χ^2^-value	*P*-value
**Male**																	
MVPA	<30 min/d	3216	613(19.1)	77.945	<0.001		586(18.2)	17.19	<0.001		532(16.5)	26.371	<0.001		576(17.9)	82.521	<0.001
	30–60 min/d	1183	104(8.8)				166(14.0)				136(11.5)				99(8.4)		
	>60 min/d	394	41(10.4)				48(12.2)				38(9.6)				28(7.1)		
Screen Time	<1 h/d	1461	122(8.4)	134.443	<0.001		133(9.1)	132.69	<0.001		89(6.1)	174.462	<0.001		116(7.9)	104.616	<0.001
	1–2 h/d	1712	255(14.9)				270(15.8)				245(14.3)				247(14.4)		
	>2 h/d	1620	381(23.5)				397(24.5)				372(23.0)				340(21.0)		
**Female**																	
MVPA	<30 min/d	5303	1075(20.3)	50.051	<0.001		1107(20.9)	81.031	<0.001		785(14.8)	3.744	0.154		1012(19.1)	52.148	<0.001
	30–60 min/d	931	103(11.1)				85(9.1)				123(13.2)				95(10.2)		
	>60 min/d	146	16(11.0)				13(8.9)				15(10.3)				12(8.2)		
Screen Time	<1 h/d	1877	219(11.7)	134.370	<0.001		187(10.0)	193.055	<0.001		124(6.6)	191.343	<0.001		199(10.6)	128.918	<0.001
	1–2 h/d	2301	408(17.7)				423(18.4)				318(13.8)				389(16.9)		
	>2 h/d	2202	567(25.7)				595(27.0)				481(21.8)				531(24.1)		
**Total**																	
MVPA	<30 min/d	8519	1688(19.8)	136.820	<0.001		1693(19.9)	90.65	<0.001		1317(15.5)	24.340	<0.001		1588(18.6)	144.078	<0.001
	30–60 min/d	2114	207(9.8)				251(11.9)				259(12.3)				194(9.2)		
	>60 min/d	540	57(10.6)				61(11.3)				53(9.8)				40(7.4)		
Screen Time	<1 h/d	3338	341(10.2)	266.906	<0.001		320(9.6)	326.156	<0.001		213(6.4)	364.911	<0.001		315(9.4)	233.727	<0.001
	1–2 h/d	4013	663(16.5)				693(17.3)				563(14.0)				636(15.8)		
	>2 h/d	3822	948(24.8)				992(26.0)				853(22.3)				871(22.8)		

Note: MVPA, moderate-to-vigorous physical activity

Table 3 shows the results of binary logistic regression analysis of different MVPA and screen time with psychological symptoms among Chinese university students. The overall results showed that, with the group of university student with MVPA > 60 min/d as the reference group, after adjusting for relevant influencing factors (Model 3), it could be seen that university students in the group with MVPA < 30 min/d (OR = 2.84,95% CI: 2.04–3.96) had a significantly increased risk of psychological symptoms (*P* < 0.001). As for the screen time, the risk of psychological symptoms was increased in universities with a screen time of 1–2 h/d (*OR* = 1.85, 95% *CI*: 1.60–2.14) compared with those with a screen time of < 1 h/d as a reference group (*P* < 0.001). And as the screen time continued to increase, university students with screen time > 2 h/d group (*OR* = 2.72,95% *CI*: 2.36–3.13) had a higher risk of developing psychological symptoms (*P* < 0.001). The results of binary logistic regression analysis of MVPA and screen time with psychological symptoms for Chinese university males and females are shown in Table 3.

**Table 3:** Binary logistic regression analysis of different MVPA and screen time with psychological symptoms among Chinese university students

Gender	Variable	Group	Model 1				Model 2					Model 3		
			OR (95% CI)	*P*-value		OR (95% CI)		*P*-value			OR (95% CI)		*P*-value	
**Male**	MVPA	>60 min/d	1.00			1.00					1.00			
		30–60 min/d	1.19(0.77 ~ 1.85)	0.426		1.28(0.82 ~ 1.98)		0.276			1.26(0.80 ~ 1.97)		0.321	
		<30 min/d	2.85(1.92 ~ 4.23)	<0.001		2.99(2.01 ~ 4.46)		<0.001			2.81(1.87 ~ 4.21)		<0.001	
	Screen Time	<1 h/d	1.00			1.00					1.00			
		1–2 h/d	1.96(1.55 ~ 2.47)	<0.001		2.00(1.58 ~ 2.54)		<0.001			2.00(1.57 ~ 2.54)		<0.001	
		>2 h/d	3.08(2.46 ~ 3.85)	<0.001		2.99(2.38 ~ 3.75)		<0.001			2.72(2.15 ~ 3.42)		<0.001	
**Female**	MVPA	>60 min/d	1.00			1.00					1.00			
		30–60 min/d	1.27(0.68 ~ 2.38)	0.457		1.15(0.61 ~ 2.17)		0.663			1.22(0.65 ~ 2.29)		0.547	
		<30 min/d	2.63(1.45 ~ 4.77)	<0.001		2.27(1.24 ~ 4.13)		0.008			2.34(1.28 ~ 4.26)		0.006	
	Screen Time	<1 h/d	1.00			1.00					1.00			
		1–2 h/d	1.72(1.43 ~ 2.06)	<0.001		1.76(1.46 ~ 2.12)		<0.001			1.77(1.47 ~ 2.13)		<0.001	
		>2 h/d	2.68(2.25 ~ 3.20)	<0.001		2.84(2.37 ~ 3.40)		<0.001			2.80(2.34 ~ 3.35)		<0.001	
**Total**	MVPA	>60 min/d	1.00			1.00					1.00			
		30–60 min/d	1.26(0.89 ~ 1.80)	0.196		1.33(0.93 ~ 1.90)		0.118			1.34(0.94 ~ 1.92)		0.109	
		<30 min/d	2.86(2.07 ~ 3.97)	<0.001		2.93(2.11 ~ 4.07)		<0.001			2.84(2.04 ~ 3.96)		<0.001	
	Screen Time	<1 h/d	1.00			1.00					1.00			
		1–2 h/d	1.81(1.57 ~ 2.09)	<0.001		1.85(1.60 ~ 2.14)		<0.001			1.85(1.60 ~ 2.14)		<0.001	
		>2 h/d	2.83(2.47 ~ 3.25)	<0.001		2.88(2.50 ~ 3.32)		<0.001			2.72(2.36 ~ 3.13)		<0.001	

Note: MVPA, moderate-to-vigorous physical activity; OR, odds ratio; 95% CI, 95% confidence intervals. Model 1 was the crude model, Model 2 adjusted for age, only child or not, father’s education, mother’s education, and SES on the basis of Model 1, and Model 3 adjusted for sleep quality, SSBs, and BMI on the basis of Model 2

**Fig. 2:**
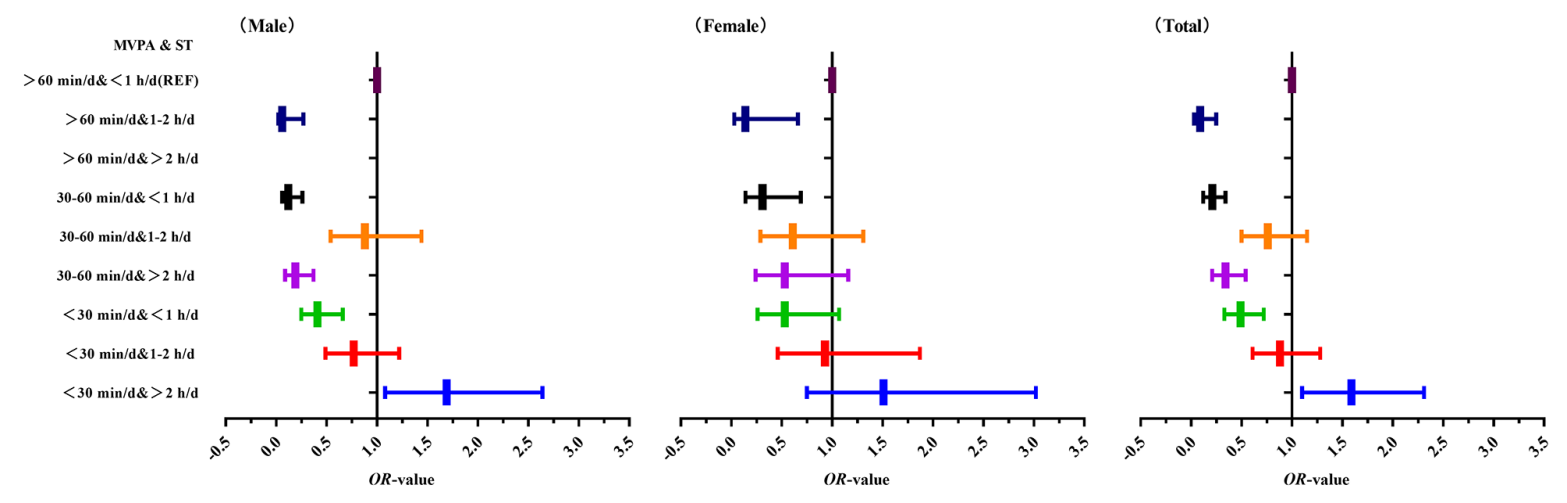
Trends in OR of MVPA and screen time with psychological symptoms among Chinese university students

Table 4 shows the results of the generalize linear model (GLM) analysis of between MVPA and screen time and psychological symptoms among Chinese university students. The overall results showed that university students with MVPA < 30 min/d and screen time > 2 h/d (*OR* = 1.59, 95% *CI*: 1.10–2.31) had the highest risk of psychological symptoms (*P* < 0.05) compared with university students with MVPA > 60 min/d and screen time < 1 h/d as the reference group. In addition, university students with MVPA > 60 min/d and screen time of 1–2 h/d (*OR* = 0.09, 95% *CI*: 0.03–0.25) had the lowest risk of psychological symptoms (*P* < 0.001). It showed that there was a significant between MVPA and screen time and psychological symptoms among Chinese university students. This shows that Chinese university students should perform MVPA for not less than 60 min a day, while controlling the duration of screen time, and screen time is controlled between 1 and 2 h a day, which has a better promotion effect on psychological health. The results of the generalize linear model analysis for different genders are shown in Table 4. The trend of OR of MVPA and screen time with psychological symptoms among Chinese university students is shown in Fig. 2.

**Table 4:** Generalize linear model (GLM) analysis of between MVPA and screen time and psychological symptoms among Chinese university students

Gender	Classification		Ordered Logistic Regression	
	MVPA	Screen Time	OR (95% CI)	*P*-value
**Male**	>60 min/d	<1 h/d	1.00	
		1–2 h/d	0.06(0.02 ~ 0.27)	<0.001
		>2 h/d	——	——
	30–60 min/d	<1 h/d	0.12(0.06 ~ 0.26)	<0.001
		1–2 h/d	0.88(0.54 ~ 1.44)	0.601
		>2 h/d	0.19(0.09 ~ 0.37)	<0.001
	<30 min/d	<1 h/d	0.41(0.25 ~ 0.66)	<0.001
		1–2 h/d	0.77(0.49 ~ 1.22)	0.272
		>2 h/d	1.69(1.08 ~ 2.64)	0.021
**Female**	>60 min/d	<1 h/d	1.00	
		1–2 h/d	0.14(0.03 ~ 0.66)	0.013
		>2 h/d	——	——
	30–60 min/d	<1 h/d	0.31(0.14 ~ 0.69)	0.004
		1–2 h/d	0.61(0.29 ~ 1.31)	0.204
		>2 h/d	0.53(0.24 ~ 1.16)	0.114
	<30 min/d	<1 h/d	0.53(0.26 ~ 1.07)	0.075
		1–2 h/d	0.93(0.46 ~ 1.87)	0.833
		>2 h/d	1.51(0.75 ~ 3.02)	0.250
**Total**	>60 min/d	<1 h/d	1.00	
		1–2 h/d	0.09(0.03 ~ 0.25)	<0.001
		>2 h/d	——	——
	30–60 min/d	<1 h/d	0.21(0.12 ~ 0.34)	<0.001
		1–2 h/d	0.76(0.50 ~ 1.15)	0.196
		>2 h/d	0.34(0.21 ~ 0.54)	<0.001
	<30 min/d	<1 h/d	0.49(0.33 ~ 0.72)	<0.001
		1–2 h/d	0.88(0.61 ~ 1.28)	0.507
		>2 h/d	1.59(1.10 ~ 2.31)	0.014

Note: MVPA, moderate-to-vigorous physical activity; OR, odds ratio; 95% CI, 95% confidence intervals. Generalize linear model (GLM) analysis was performed with age, only child or not, father’s education, mother’s education, SES, sleep quality, SSBs, and BMI as covariates

## Discussion

The results of this study showed that the proportion of Chinese university students with MVPA > 60 min/d was 4.8%, and this result was low compared with the results of related studies [34]. The World Health Organization recommends that adolescents should engage in no less than 60 min of MVPA per day. The results of this study indicate that physical inactivity is particularly problematic among Chinese university students. The study confirmed that only 20.0% of adults aged 18–29 years in the United States meet the recommended standard for physical activity, which is not less than 60 min of moderate to vigorous physical activity per day [34]. The proportion of Hong Kong, China, university students meeting the MVPA standard is low, with an average of only 38.77 min per day [35]. However, the percentage of U.S. college students with an MVPA is also relatively low [36]. This indicates that the problem of insufficient physical activity among Chinese university students is particularly pronounced. The results of this study also showed that the proportion of Chinese university students with screen time > 2 h/d was 34.2%. This indicates that a significant proportion of university students’ screen time is in the over-standard range. The proportion of Indian adolescents with screen time > 120 min/d was 85.0%(34). In addition, there are studies that show that 16.8% of teenagers in China have more than 2 h of screen time/day [27]. Another survey of Chinese university students showed that 20.8% of university students in China had screen time > 120 min/d and that increased screen time was associated with increased risk of depression [37]. This also shows that it is necessary to take measures to control the continuous prolongation of screen time among university students in order to better promote their health. This study also showed that the detection rate of psychological symptoms among Chinese university students was 16.3%, with a higher rate among female students (17.5%) than male students (14.7%), a result that is lower than the global study of 34% detection rate of psychological symptoms [38]. It may be related to the different groups, time and questionnaires investigated in different studies, resulting in some differences between the results.

The results of this study also showed that compared to the group of university students with MVPA > 60 min/d, after adjusting for relevant influencing factors, it was seen that university students in the group with MVPA < 30 min/d (OR = 2.84) were at significantly increased risk of psychological symptoms. A study of adolescents also confirmed that those with higher MVPA levels had lower levels of depression and anxiety, consistent with the findings of this study [39]. A study of Chinese adolescents also showed that adolescents who exercised < 30 min/d (OR = 1.62) had a significantly increased risk of psychological symptoms [27]. Another study also confirmed that adequate physical activity and a proper diet have a significant impact on the prevention of depression [40]. Another study also confirmed that adequate physical activity plays a protective role in preventing depression in adolescents [41]. For specific reasons, on the one hand, the increase in adolescents’ MVPA is often accompanied by an increase in physical activity time, which has a positive impact on mental health through communication and exchange with peers during physical activity, thus releasing their own stress. On the other hand, a study confirmed that muscle contraction releases chemicals into the bloodstream, and that a myocytokine can cross the blood-brain barrier and enter the brain [40]. This myocytokine has a positive effect on the regulation of brain functions, including mood, learning, athletic ability, improving brain immunity, and also acts as an antidepressant [40]. The results of this study better illustrate the effect of increased physical activity in physical exercise on mental health [42].

In terms of video screen behavior, the results of this study showed that university students with a screen time of 1–2 h/d (OR = 1.85) were at increased risk of psychological symptoms compared to those with a screen time of < 1 h/d as a reference group. As the screen time continued to increase, university students with screen time > 2 h/d (OR = 2.72) had a higher risk of psychological symptoms. A study confirmed that there is a strong association between daily electronic device use and longer sedentary time in adolescents, and that longer sedentary time leads to obesity and a range of psychological problems, which have a negative impact on their health [43]. A cohort study of young people in the UK showed an increased risk of depression among those who spent 1–2 h (OR = 1.12) and more than 3 h (OR = 1.35) on the computer at weekends compared to those who spent < 1 h on the computer at weekends [44]. A study of Chinese adolescents showed that adolescents with screen time > 2 h/day had a significantly increased risk of anxiety (OR = 1.38) and depression (OR = 1.55) and psychiatric disorders (OR = 1.49) compared to those with screen time ≤ 2 h/day. After further adjustment for gender, age, residential background, body mass index, household economy, sleep quality, smoking, and alcohol consumption, the results did not change significantly, and overall, screen time had a negative but relatively small effect on mental health [45]. This result suggests that appropriate screen time should be maintained to better ensure healthy mental development. In the future, appropriate video screen time should be secured in the implementation of the intervention to achieve better intervention results.

Most of the previous studies analyzed the association between MVPA and screen time in terms of a single aspect of the association that exists with psychological symptoms. The present study further analyzed the association between of MVPA and screen time and psychological symptoms. The results showed that university student with MVPA < 30 min/d and screen time > 2 h/d (*OR* = 1.59) had the highest risk of psychological symptoms. In addition, university students with MVPA > 60 min/d and screen time of 1–2 h/d (*OR* = 0.09) had the lowest risk of psychological symptoms. This result also suggests that future mental health interventions for university students should ensure adequate time for MVPA and control the screen time within a reasonable range, maintaining 1–2 h of screen time per day. Just as the results of the study, the reasonable use of online tools by adolescents can promote psychological and spiritual communication and exchange, facilitate the establishment of offline friendships, and play a positive role in relieving psychological stress and promoting the development of mental health [18]. This result also suggests that in the current era of highly developed electronic information, maintaining a reasonable amount of screen time plays a positive role in promoting mental health and should be given attention and importance in the process of future psychological interventions.

There are some strengths and limitations of this study. Strengths: First, to the best of our knowledge, this is the first survey of physical activity, screen time and psychological symptoms among university students in six regions of China, and the sample is somewhat representative. Secondly, this study used the effect analysis of MVPA and screen time to analyze the relationship with psychological symptoms, which can provide a more in-depth understanding of the factors affecting psychological symptoms and provide reference and help for later mental health interventions. However, there are some limitations in this study. On the one hand, this study is a cross-sectional study, which can only understand the correlation between them, but not the causal association. On the other hand, the covariates investigated in this study were limited, and more covariates, such as eating behavior and waist circumference, should be included in future studies.

## Conclusion

This study was the first to analyze the association between MVPA and screen time and psychological symptoms among Chinese university students. The results showed that MVPA and screen time were associated with psychological symptoms, and there were also significant between MVPA and screen time and psychological symptoms among Chinese university students. The results of this study showed that the best state for maintaining mental health is based on ensuring MVPA > 60 min/d, screen time is not the shorter the better, but a certain amount of screen time is necessary, and screen time controlled at 1–2 h/d is the most appropriate, that is, the lowest risk of psychological symptoms. In view of the results of this study, it is recommended that Chinese university students should perform MVPA for not less than 60 min per day, and at the same time control the duration of screen time, with screen time controlled between 1 and 2 h per day, which has a better promotion effect on maintaining psychological health. In addition, the findings of this study may also provide some reference and lessons for the education and administrative departments in the future to formulate more reasonable education policies and family education strategies.

## Data Availability

The datasets analysed during the current study are not publicly available due to protect the privacy of participants, the questionnaire data will not be disclosed to the public but are available from the corresponding author on reasonable request.
